# Dr. Emily Blackwell

**Published:** 1910-09-17

**Authors:** 


Dr. Emily Blackwell, born at Bristol, England, in 1826, has died at Maine, U.S.A. She was, says the *Times*, one of the earliest women physicians in America, and founded the first women's hospital. Her sister was Dr. Elizabeth Blackwell, whose death in her ninetieth year took place last May. Dr. Emily was five years younger than her sister, who had the distinction of being the first fully-qualified woman doctor. She graduated at the Western Reserve University in 1854, three years after her sister had begun practice in New York. The two sisters organised the Women's Medical College of New York Infirmary, of which Dr. Emily was dean, and founded the New York Infirmary for Women and Children, the first women's hospital in America.

